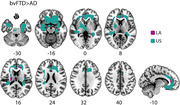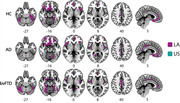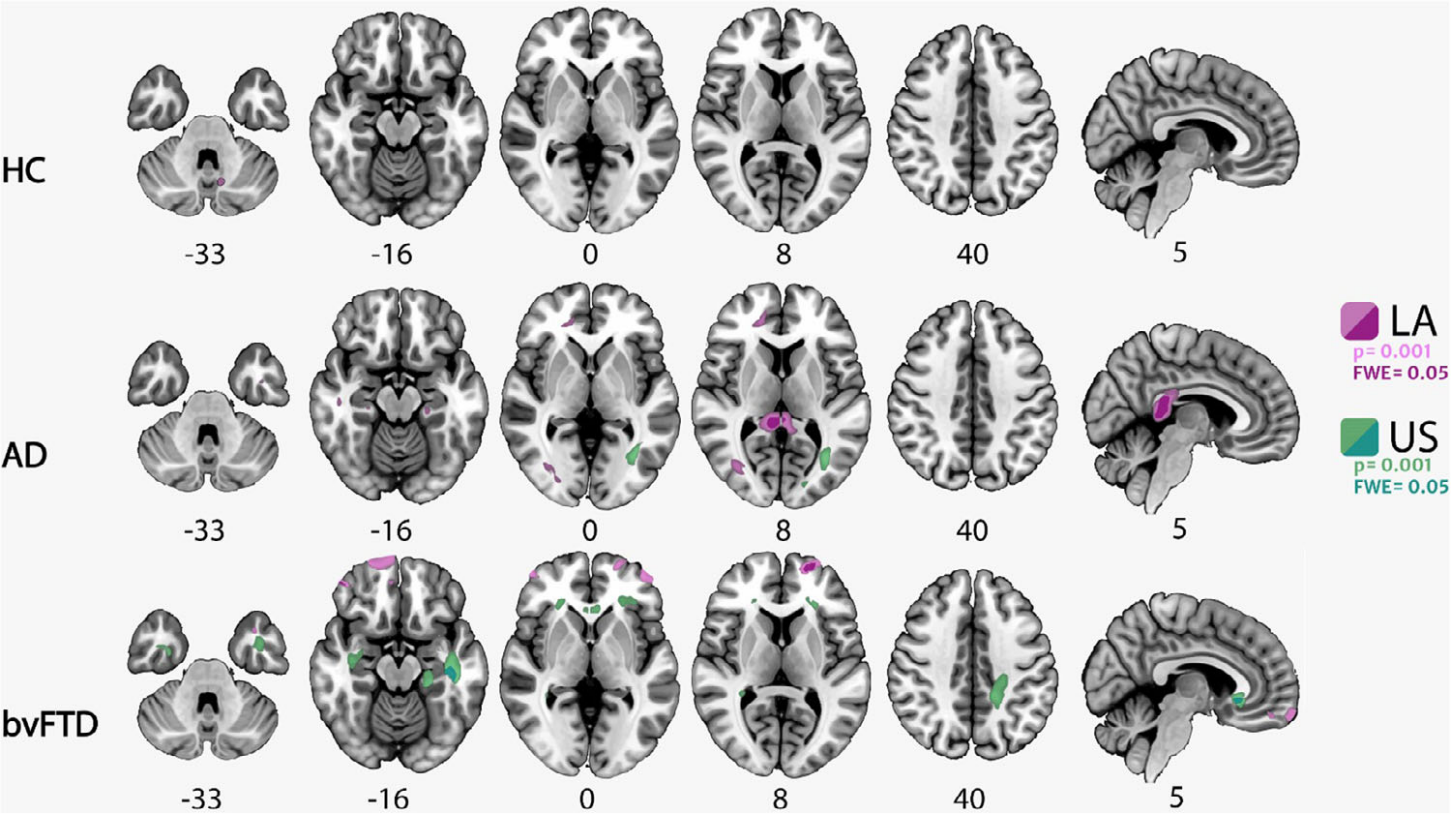# White matter hyperintensities in AD and bvFTD patients from Underrepresented countries

**DOI:** 10.1002/alz.085189

**Published:** 2025-01-09

**Authors:** Florencia Altschuler, Veronica Canziani, Matias Fraile‐Vazquez, Agustín Ibáñez, Cecilia Gonzalez Campo

**Affiliations:** ^1^ Cognitive Neuroscience Center (CNC), Universidad de San Andres, Buenos Aires Argentina; ^2^ Universidad de Buenos Aires, Buenos Aires Argentina; ^3^ Global Brain Health Institute, Trinity College Dublin, Dublin Ireland; ^4^ Trinity College, Dublin Ireland; ^5^ Latin American Brain Health Institute (BrainLat), Universidad Adolfo Ibáñez, Santiago Chile; ^6^ Cognitive Neuroscience Center (CNC), Universidad de San Andrés, Buenos Aires Argentina; ^7^ CONICET, Buenos Aires Argentina; ^8^ Cognitive Neuroscience Center (CNC), Universidad de San Andrés, Buenos Aires, Buenos Aires Argentina

## Abstract

**Background:**

White matter hyperintensities (WMH) are very common brain MRI signal abnormalities linked to age, small vessel cerebrovascular disease, cognitive impairment, and dementia. Despite extensive research on WMH in Alzheimer's disease (AD), their prevalence in behavioral variant Frontotemporal dementia (bvFTD) remains less explored. Additionally, Latin American countries (LA) exhibit a higher prevalence of cerebrovascular disease due to distinct demographic, socioeconomic, cultural, and ethno‐racial factors. However, limited research on WMH exists in this context. Our aim was to characterize the WMH burden and its relationship with neurodegeneration and cognition in healthy aging and dementia subjects from LA in comparison to the United States (US).

**Methods:**

This cross‐sectional multicenter study involved 638 LA and 355 US subjects from the ReDLat consortium, including healthy controls (HC), bvFTD, and AD patients. Participants underwent brain MRI and neuropsychological assessments. Voxel‐based morphometry analysis was performed to calculate WMH load and distribution from T2‐FLAIR images, and gray matter (GM) atrophy from T1 images. To assess the differences in the spatial distribution of WMH, two sample t‐tests were run between HC and neurodegenerative groups (AD and bvFTD) for each region (LA and US). The association between total load of WMH with regional GM volume and the association between cognitive performance (MMSE) with tract‐specific WMH load was tested via voxel‐wise regression analyses. In all analysis significance was set at p<0.05 family‐wise error‐corrected for multiple comparisons with a cluster extent threshold of 50 voxels. Age, years of education, sex, TIV and scanner were included as covariates of no interest.

**Results:**

bvFTD exhibited higher WMH load than AD in both regions (LA and US, Figure 1). Notably, the association between WMH burden and GM atrophy was substantially stronger for all LA groups (bvFTD, AD, and HC) compared to the US (Figure 2). Additionally, an association between WMH burden and cognitive impairment (MMSE) was found in all pathological groups (Figure 3).

**Conclusion:**

This study highlights regional and disease‐specific effects of WMH burden in brain atrophy and cognition, emphasizing the importance of region‐adapted dementia research for a comprehensive understanding of these disparities.